# Evaluation of *Legionella* Air Contamination in Healthcare Facilities by Different Sampling Methods: An Italian Multicenter Study

**DOI:** 10.3390/ijerph14070670

**Published:** 2017-06-22

**Authors:** Maria Teresa Montagna, Osvalda De Giglio, Maria Luisa Cristina, Christian Napoli, Claudia Pacifico, Antonella Agodi, Tatjana Baldovin, Beatrice Casini, Maria Anna Coniglio, Marcello Mario D’Errico, Santi Antonino Delia, Maria Grazia Deriu, Marco Guida, Pasqualina Laganà, Giorgio Liguori, Matteo Moro, Ida Mura, Francesca Pennino, Gaetano Privitera, Vincenzo Romano Spica, Silvia Sembeni, Anna Maria Spagnolo, Stefano Tardivo, Ida Torre, Federica Valeriani, Roberto Albertini, Cesira Pasquarella

**Affiliations:** 1 Department of Biomedical Science and Human Oncology, University of Bari “Aldo Moro”, Piazza G. Cesare 11, 70124 Bari, Italy; osvalda.degiglio@uniba.it (O.D.G.); pacifico.cla@gmail.com (C.P.); 2 Department of Health Sciences, University of Genoa, Via Pastore 1, 16132 Genova, Italy; cristinaml@unige.it (M.L.C.); am.spagnolo@unige.it (A.M.S.); 3 Department of Medical and Surgical Sciences and Translational Medicine, Sapienza University of Roma, Via di Grottarossa 1035, 00189 Roma, Italy; christian.napoli@uniroma1.it; 4 Department of Medical and Surgical Sciences and Advanced Technologies “GF Ingrassia”, University of Catania, Via Sofia 87, 95123 Catania, Italy; agodia@unict.it (A.A.); ma.coniglio@unict.it (M.A.C.); 5 Department of Cardiac, Thoracic and Vascular Sciences, Hygiene and Public Health Unit, University of Padova, Via Loredan 18, 35131 Padova, Italy; tatjana.baldovin@unipd.it; 6 Department of Translational Research and New Technologies in Medicine and Surgery, University of Pisa, Via San Zeno 35/39, 56127 Pisa, Italy; beatrice.casini@med.unipi.it (B.C.); gaetano.privitera@med.unipi.it (G.P.); 7 Department of Biomedical Sciences and Public Health, Polytechnic University of Marche, via Tronto, 10/a Torrette di Ancona, 60020 Ancona, Italy; derrico@univpm.it; 8 Department of Biomedical Science and Morphological and Functional Images, University of Messina, Via C.Valeria snc, 98125 Messina, Italy; adelia@unime.it (S.A.D.); plagana@unime.it (P.L.); 9 Department of Biomedical Science-Hygiene Section, University of Sassari, Via Padre Manzella 4, 07100 Sassari, Italy; mariagrazia.deriu@aousassari.it (M.G.D.); idamura@uniss.it (I.M.); 10 Department of Biology, University of Napoli “Federico II”, Via Cinthia 26, 80126 Napoli, Italy; marco.guida@unina.it; 11 Department of Movement Sciences and Wellbeing, University “Parthenope”, Via Medina 40, 80133 Napoli, Italy; giorgio.liguori@uniparthenope.it; 12 IRCCS San Raffaele Scientific Institute, Via Olgettina 60, 20132 Milano, Italy; moro.matteo@hsr.it; 13 Department of Public Health, University of Napoli “Federico II”, Via S.Pansini 5, 80131 Napoli, Italy; francesca.pennino@unina.it (F.P.); ida.torre@unina.it (I.T.); 14 Department of Movement, Human and Health Sciences, Public Health Unit, University of Roma “Foro Italico”, P.zza Lauro De Bosis 6, 00135 Roma, Italy; vincenzo.romanospica@uniroma4.it (V.R.S.); valerianife@yahoo.it (F.V.); 15 Department of Diagnostic and Public Health, University of Verona, Strada le Grazie 8, 37134 Verona, Italy; silvia.sembeni@univr.it (S.S.); stefano.tardivo@univr.it (S.T.); 16 Department of Medicine and Surgery, University of Parma, Medical Immunology Unit, University Hospital of Parma, Via Gramsci 14, 43126 Parma, Italy; roberto.albertini@unipr.it; 17 Department of Medicine and Surgery, University of Parma, Via Volturno 39, 43125 Parma, Italy; ira.pasquarella@unipr.it

**Keywords:** Coriolis^®^μ, Surface Air System, settle plates, index microbial air

## Abstract

Healthcare facilities (HF) represent an at-risk environment for legionellosis transmission occurring after inhalation of contaminated aerosols. In general, the control of water is preferred to that of air because, to date, there are no standardized sampling protocols. *Legionella* air contamination was investigated in the bathrooms of 11 HF by active sampling (Surface Air System and Coriolis^®^μ) and passive sampling using settling plates. During the 8-hour sampling, hot tap water was sampled three times. All air samples were evaluated using culture-based methods, whereas liquid samples collected using the Coriolis^®^μ were also analyzed by real-time PCR. *Legionella* presence in the air and water was then compared by sequence-based typing (SBT) methods. Air contamination was found in four HF (36.4%) by at least one of the culturable methods. The culturable investigation by Coriolis^®^μ did not yield *Legionella* in any enrolled HF. However, molecular investigation using Coriolis^®^μ resulted in eight HF testing positive for *Legionella* in the air. Comparison of *Legionella* air and water contamination indicated that *Legionella* water concentration could be predictive of its presence in the air. Furthermore, a molecular study of 12 *L. pneumophila* strains confirmed a match between the *Legionella* strains from air and water samples by SBT for three out of four HF that tested positive for *Legionella* by at least one of the culturable methods. Overall, our study shows that *Legionella* air detection cannot replace water sampling because the absence of microorganisms from the air does not necessarily represent their absence from water; nevertheless, air sampling may provide useful information for risk assessment. The liquid impingement technique appears to have the greatest capacity for collecting airborne *Legionella* if combined with molecular investigations.

## 1. Introduction

*Legionella* is a ubiquitous intracellular microorganism present in both natural (e.g., rivers, lakes, and ponds) and artificial (e.g., potable water systems, taps, faucets, showers, cooling towers and fountains) aquatic environments. This microorganism grows at temperatures of 25 °C–50 °C, especially if the water is stagnant and rich in sediments, and is responsible for various clinical manifestations, including the pneumonia known commonly as Legionnaires’ disease (LD) [1]. The genus *Legionella* includes 59 different bacterial species and 70 serogroups (sg). Although *Legionella pneumophila* (Lpn) sg 1 and sg 6 are the main causes of disease, other species such as *L. cardiac* and *L*. *nagasakiensis* have recently been associated with cases of legionellosis [2].

Healthcare facilities (HF) represent an at-risk environment for LD transmission because they frequently have old plumbing systems and contain medical devices used by immunocompromised patients [1]. The European surveillance reported 5851 cases of LD in 2013 by 28 member states, with 8% of cases linked to HF [3]. In Italy, amongst the 1569 cases reported in 2015 (incidence 25.8 cases per million inhabitants), 83 (5.3%) were admitted to hospital [4].

Legionnaires’ disease normally occurs after inhalation of aerosols produced from contaminated water sources. Although one case of human-to-human transmission has recently been reported [5], there are still many doubts regarding whether this can actually occur; therefore, it can be presumed that the environment is the main source of infection. 

Most documents regarding the control and prevention of LD provide recommendations concerning sampling of different environmental matrices (water, fouling, deposits, etc.), but not air [1,2]. The control of water is mainly preferred based on standardized sampling, which is able to trace the source of infection. However, some authors highlight that air sampling could be combined with water systems surveillance as a useful tool for preventive legionellosis [6]. Nevertheless, the presence of *Legionella* in air samples, both indoors [7,8] and outdoors [9,10], highlights the difficulties in detecting *Legionella*.

Active and passive methods are generally used to evaluate microbial air contamination [11]. The Surface Air System (SAS), which is one of the most commonly used active sieve air samplers, results in air impacting a solid surface. The impinger method is a method of active air sampling onto liquid medium that allows the detection of airborne microorganisms by culture and molecular investigations. Passive sampling, which measures the rate at which viable particles settle on surfaces, is standardized according to the index of microbial air (IMA) contamination [12]. These sampling methods are also utilized to evaluate *Legionella* indoor air contamination [7,13,14,15,16,17,18]; however, no specific protocols for their use in *Legionella* air sampling have been developed to date. 

In previous studies [16,17], the Italian Study Group on Hospital Hygiene (GISIO) of the Italian Society of Hygiene, Preventive Medicine, and Public Health (SItI), in collaboration with the Italian Association of Aerobiology (AIA) compared the use of SAS impacting onto solid substrate to the use of settling plates for the evaluation of *Legionella* air contamination. The low number of positive results has encouraged repeated experiments using the cyclone sampler Coriolis^®^μ, an active air sampler on liquid medium, to quantify *Legionella* in bioaerosols by both culture-based and molecular investigations [19,20,21,22]. 

Based on our previous experience [7,14,15,16,17,23,24,25,26], the GISIO-SItI group and AIA group, in collaboration with the Italian Multidisciplinary Society for the Prevention of Infection in Healthcare Organizations (SIMPIOS), promoted this multicenter study to (i) evaluate *Legionella* air contamination in HF using different air sampling methods, (ii) determine a molecular method for comparison between the same Lpn strains isolated from the air and water samples, (iii) evaluate *Legionella* concentrations in water related to its presence in the air; (iv) contribute to a definition of a standardized sampling protocol to detect *Legionella* airborne contamination.

## 2. Materials and Methods

Eleven HF from eight Italian regions (Liguria, Veneto, Tuscany, Campania, Lazio, Apulia, Sardinia and Sicily) were voluntarily enrolled in the study after one bathroom was identified as having a water supply contaminated with >1000 colony-forming units (CFUs)/L of *Legionella pneumophila* (Lpn) and *Legionella species* (Lspp). Air contamination was assessed by active and passive sampling within a period of eight hours (from 9 a.m. to 5 p.m.). During the air sampling time, the hot tap water of the selected water supply was sampled three times for cultural investigation. 

For the first isolation of *Legionella* in the culturable investigation, plates containing Glycine–Vancomycin–Polymyxin–Cycloheximide medium (GVPC, Liofilchem Srl, Teramo, Italy) were incubated at 36 °C for 10 days in a humid environment under 2.5% CO_2_. Suspect colonies were then subcultured on charcoal yeast extract medium (Liofilchem Srl, Teramo, Italy) without L-cysteine and Buffered Charcoal Yeast Extract medium (BCYE; Liofilchem Srl, Teramo, Italy) with L-cysteine. Colonies growing only on BCYE agar plates were considered to belong to the *Legionella* genus and therefore subjected to identification using a latex agglutination test with polyvalent (Oxoid Spa, Milan, Italy) and monovalent antisera (Biogenetics Srl, Tokyo, Japan). Molecular analysis was performed by real-time PCR and isolates of *Legionella* from air and water were compared by the sequence-based typing (SBT) method.

### 2.1. Air Sampling

Air contamination was assessed by active sampling using the Surface Air System (SAS, PBI International, Milan, Italy) and Coriolis^®^μ (Bertin Technologies, Montigny le Bretonneux, France), and by passive sampling using settling plates. The SAS and Coriolis^®^μ samplers were located 1 m from the floor and 30 cm from the tap. Every 12 min and during the two minutes of flushing water (overall, five flushing water/h), 200 L of air was aspirated for a total of 1000 L/h. The plate (used for SAS) and the cone containing 15 mL of liquid substrate (0.005% Triton X-100employed for Coriolis^®^μ) were changed at the end of each sampling hour. Overall, 40 aspirations were performed on a total of eight plates (five aspirations/plate/h) and eight cones (five aspirations/cone/h), respectively. For SAS, the number of CFUs was adjusted using the conversion table provided by the manufacturer, and the value was expressed in CFU/m^3^.

For Coriolis^®^μ, after aspiration, the volume of liquid substrate was measured and annotated because a portion is assumed to be lost by evaporation. The remaining fluid [V_buffer after sampling_] was aseptically transferred to a sterile container for culture-based and molecular investigations. 

For isolation of *Legionella* in the culturable investigation, the liquid sample was vortexed for 5 to 10 min, after which 0.5 mL [V_plated aliquot_] of the original sampling solution and of 1:10 dilutions were plated on selective agar for *Legionella*. The average numbers of colonies (CFU) were used to calculate the total culturable airborne *Legionella* using the following equation [27,28]:

$$\mathrm{CFU}/m^{3}\;=\frac{\mathrm{cfu}}{V_{\mathrm{plated}\;\mathrm{aliquote}\;}\left[\mathrm{mL}\right]}\;\times \;\frac{\mathrm{dilution}\;\mathrm{factor}\;\times V_{\mathrm{buffer}\;\mathrm{after}\;\mathrm{sampling}\;}\left[\mathrm{mL}\right]}{V_{\mathrm{air}\;\mathrm{sample}}\left[m^{3}\right]}$$

After cultural investigations, the volume of the remaining liquid collection of Coriolis^®^μ was adjusted to 100 mL with distilled sterile water for molecular analysis by real-time PCR (Polymerase Chain Reaction). DNA was then extracted using an Aquadien Kit (BioRad, Hercules, CA, USA) according to the manufacturer’s protocol. Next, 5 μL of extracted DNA was used for qPCR (quantitative PCR) with iQ Check Quanti *Legionella* pneumophila and *Legionella* species kits (BioRad, Hercules, CA, USA). A CFX 96-deep well real-time detection System (BioRad, Hercules, CA, USA) and the CFX Manager Software (BioRad, Hercules, CA, USA) were utilized to determine the number of genomic units per well (GU/well). Total GU was calculated by the obtained GU/well × 132 (a factor taking into account the volumes analyzed, purified and subjected to PCR).

Passive sampling was performed using settle plates 90 mm in diameter, which were left exposed to the air for 1 h at 1 m above the floor, to determine the index of microbial air contamination (IMA) [12]. Specifically, two plates/h were placed at 30 cm from the selected tap water. The result was reported as the average of values measured on 16 plates/8 h and expressed as CFU/plate.

### 2.2. Water Sampling

The hot tap water of the selected water supply was sampled three times in routine flushing conditions: T0 = before starting the first air sampling; T1 = after 4 h; and T2 = 8 h after the end of the air sampling, according to the procedures reported in the Italian Guidelines for the Prevention and Control of Legionellosis [2].

### 2.3. Comparison of Legionella in Water and in the Air

A molecular study was conducted to compare *Legionella pneumophila* strains isolated from air (one strain for each different serogroup isolated by all culture methods) and water samples (one strain for each different serogroup isolated). Genotyping was performed via the standard sequence-based typing (SBT) method of the European Working Group for *Legionella* Infections (EWGLI) using seven genes (flaA, pilE, asd, mip, mompS, proA, and neuA) [29,30].

For each gene sequence, a distinct allele number was assigned through the EWGLI–SBT database for *Legionella pneumophila*. The combination of these allele numbers defines an allelic profile to which a sequence type (ST) is attributed using the EWGLI–SBT database. For the strains from which neuA could not be amplified, primers targeting neuAh were used, as suggested by the European Study Group for *Legionella* Infections [31]. The neuAh gene, which is present in some Lpn non-sg 1 strains, is functionally equivalent to the neuA gene of the Lpn subsp. pneumophila Philadelphia-1 strain.

### 2.4. Statistical Analysis

Quantitative values of *Legionella* in the air were reported as median and interquartile ranges. For each hospital, the presence of *Legionella* in water was calculated as the mean of three values obtained during the sampling times (T0–T1–T2). Box plots was generated to describe the distribution of *Legionella* water concentrations and to represent the minimum and maximum values, medians and quartiles.

To compare *Legionella* water concentrations with *Legionella* in the air, the HF were divided into two groups. The positive group (PG) included HF that tested positive for *Legionella* in the air by at least one culture-based method, while the negative group (NG) included HF that were never positive for *Legionella* by culture-based methods.

The presence of *Legionella* in the air based on molecular investigations was calculated as the median of eight values obtained during the sampling times, and expressed in genomic units (GU)/100 mL.

Due to the non-normal distribution of data (tested by the Shapiro–Wilk test), the non-parametric Mann–Whitney U test was used to compare the median values of (i) *Legionella* in water (CFU/L) between PG and NG, and (ii) *Legionella* in the air as determined by molecular methods (GU/100 mL) between PG and NG. Moreover, when only the PG was considered, the non-parametric Mann–Whitney U test was used to assess whether the molecular investigation was able to show an increase of *Legionella* in the air during the hours of positive sampling by the culture-based method. 

The ability of *Legionella* water concentration to predict the presence of *Legionella* in the air was evaluated using receiver operating characteristic (ROC) analysis which plots the sensitivity (or true-positive fraction) and “one minus specificity” (or false-positive fraction): the higher the area under the curve, the higher the accuracy. Additionally, the best cutoff for *Legionella* water concentration was identified through the Youden’s index, which maximizes the difference between true and false positive results. 

Furthermore, a two-sample test of proportions (Z-test) was used to assess differences in positive results between all culture-based air sampling methods (SAS, settle plates and Coriolis^®^μ) and the molecular investigation using Coriolis^®^μ. 

The level of agreement among all sampling air methods (defined as the inter-agreement) was measured considering only the four HF in which *Legionella* air contamination was detected by at least one of the culture-based methods. Inter-agreement between active SAS sampling and settle plates was assessed by Cohen’s kappa coefficient (0 < *K* < 1), which takes into account the degree of concordance occurring by chance. Considering the active molecular sampling by Coriolis^®^μ as the “reference method of the air presence of *Legionella*”, the Cohen’s kappa coefficient was used to identify which of the other two methods (settle plates vs. SAS) reported the highest inter-agreement. 

The statistical software STATA 12 was used for all statistical analyses and *p* < 0.05 was considered significant.

## 3. Results

### 3.1. Legionella in the Air

The presence of *Legionella* in the air by culturable and molecular investigations is shown in Table 1. Air contamination was found in four out of 11 HF (36.4%) by at least one of the culture-based methods. Specifically, two HF were found to be positive by both SAS (1 CFU/m^3^, Lpn sg 10; 1 CFU/m^3^, Lpn sg 1, respectively) and settle plates (1 CFU/plate, Lpn sg 10; 2.25 CFU/plate, Lpn sg 1 + 7, respectively); one was positive only by SAS (1 CFU/m^3^, Lpn sg 1), and one only by settle plates (2 CFU/plate, Lpn sg 3). The cultural investigation by Coriolis^®^μ did not yield *Legionella* in any enrolled HF. Molecular investigation by Coriolis^®^μ resulted in eight HF being positive for *Legionella* in the air with different median values among positive HF (Table 1).

### 3.2. Comparison of Different Air Sampling Investigations

The molecular investigation by Coriolis^®^μ revealed a larger number of air samples positive for *Legionella* than any of the culture-based methods (*p* < 0.05). Of the eight HF in which *Legionella* was found by molecular methods, four also tested positive by at least one of the culture-based sampling systems (Table 2).

The inter-agreement between the SAS and settle plates sampling was moderate (Cohen’s kappa = 0.55). Additionally, when the results of the culture-based investigations were compared with those of the molecular-based investigation, the inter-agreement was fair for both SAS and settle plate sampling (Cohen’s kappa = 0.25). Because of the low number of air positive HF, it was not possible to determine the significance of the inter-agreement.

The presence of *Legionella* air contamination at different hours was discontinuous in the culture-based investigation, whereas it was continuous based on the results of the molecular investigation (Table 2). Two of the four HF (HF 7 and 11) showed *Legionella* presence in the air by only one of the culture-based methods, while the remaining two HF (i.e., HF 1 and 5) showed *Legionella* presence upon analysis by both active sampling and settle plates.

The molecular analysis of the *Legionella* concentration in the air showed that median value did not differ significantly between PG and NG (*p* > 0.05). When only PG was considered, there was no increase in airborne *Legionella* during the hours of sampling for which positive results were obtained by the culture-based methods (*p* > 0.05).

### 3.3. Legionella in Water

The presence of *Legionella* in water based on culturable investigations is shown in Table 1. Using this method, all 11 HF were confirmed to be positive for *Legionella* in water samples. Overall, Lpn sg 3 was the most frequently isolated serogroup (45.4%), detected in five HF (facilities in Lazio, Liguria, Veneto [two locations], and Sicily). Lpn sg 1 was found in Campania (two locations) (18.2%), while Lpn sg 7 was found in Sardinia (9.1%), Lpn sg 10 was found in Apulia (9.1%), Lpn sg 1 and sg 6 were found in Tuscany (9.1%), and Lpn sg 1, sg 7 and sg 3 were found in Sicily (9.1%).

### 3.4. Comparison between Legionella in Water and in the Air

**Figure 1:**
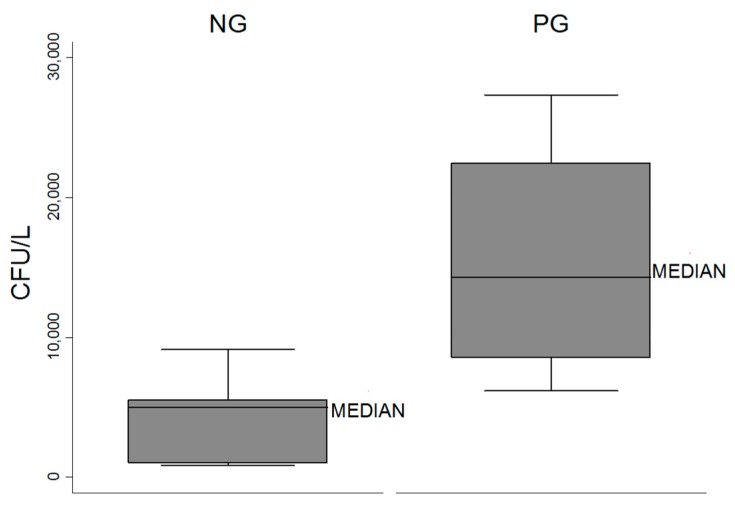
Distribution of daily mean values of *Legionella* in water of PG^1^ and NG^2^. ^1^ = positive group by at least one of the culture-based methods; ^2^ = negative group by at least one of the culture-based methods.

**Figure 2:**
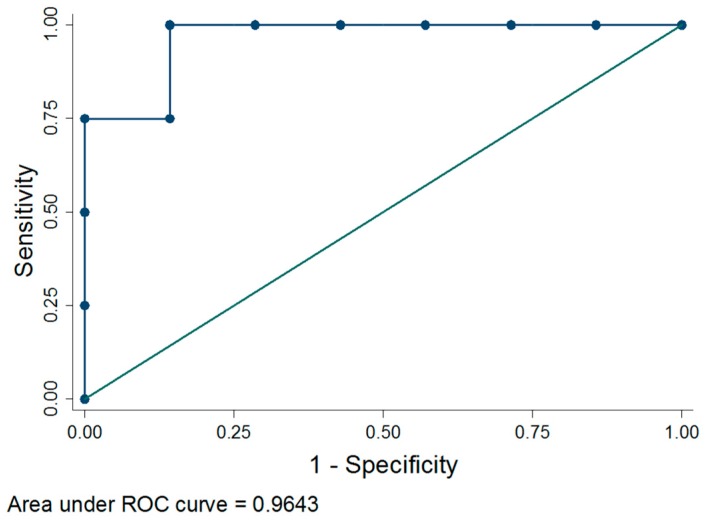
Receiver operating characteristic (ROC) curve of *Legionella* water concentration to discriminate between presence and absence of *Legionella* in the air by at least one culture-based method.

Three (HF 1, 5 and 11) of the four PG reported the highest daily mean values of *Legionella* in water (Table 1). The median in PG (14,283.33 CFU/L) was higher than in NG (4966.67 CFU/L) (*p* < 0.05, Figure 1). According to these results, the presence of *Legionella* in the air could be predicted by its concentration in water (area under ROC curve = 0.964) (Figure 2). In our study, the best cutoff result observed was 6166.67 CFU/L; this value is predictive of *Legionella* presence in the air 90.91% of the time.

The serogroups found in the air were the same as those found in the water samples, with the exception of two HF (5 and 7) that were found to be positive for Lpn sg 1 + 7 and for Lpn sg 1 both in the air and water, as well as Lpn sg 3 and Lpn sg 6, which were found only in water, respectively (Table 1).

A molecular study of the 12 Lpn strains (seven from air and five from water samples) was also conducted. A match between the *Legionella* strains from the air and water samples was confirmed by SBT for three out of four PG (HF 1, 5 and 11). In particular, ST1 (allelic profile 1, 4, 3, 1, 1, 1, 1) + ST 1919 (allelic profile 16, 21, 12, 19, 31, 21, 1) was found simultaneously in the air and water samples of HF No. 5, while ST93 (allelic profile 3, 10, 1, 28, 14, 9, 13) was isolated from the air and water samples of HF No. 11. In HF No. 1, which is located in Apulia, a new ST was isolated and typed from the air and water samples. This strain had the allelic profile 2, 10, 3, 28, 13, 4, 207 and was included in the EWGLI–SBT database and identified as ST2190.

In HF No. 7, the allelic profile of strains from air and water samples differed, with ST269 (allelic profile 7, 10, 17, 3, 13, 11, 11) being found from the air and ST657 (allelic profile 2, 10, 3, 3, 21, 4, 11) from water samples. Isolates identified in this study (ST1, ST1919, ST2190, ST93, ST269 and ST657) showed different strings of individual allele numbers, with ST2190 showing the same flaA of ST657, the same pil E allele of ST93 and ST269 and ST657, the same asd allele of ST1 and ST657, the same mip allele of ST93, the same mompS allele of ST269, and the same proA allele of ST657.

## 4. Discussion

*Legionella* air contamination was found in 36.4% of enrolled HF by at least one of the employed culture-based methods according to a previous study [16]. The introduction of another air sampler (Coriolis^®^μ) did not increase the number of positive results obtained by culture-based methods [32]. By contrast, Coriolis^®^μ showed a higher sensitivity for *Legionella* when the molecular-based method was used than when the culture-based method was applied. Some authors [20] have reported that microbial stress is influenced by the medium used and the process of liquid collection during bioaerosol collection by Coriolis^®^μ. We used a liquid collection containing Triton X-100 (0.005%), of which 0.5 mL was inoculated onto selective agar medium according to the manufacturer’s instructions. However, other authors [28,33] have obtained a larger number of positive results using a selective treatment process only for *Legionella* detection.

With regard to the comparison between active and passive methods, although there was a low number of positive results, moderate inter-agreement between the two methods was observed. Previous studies have confirmed a correlation between the two sampling methods for microbial air contamination, but not specifically for *Legionella* [13,14,25].

In our study, the *Legionella* water concentration influenced the detection of *Legionella* in the air, which is in accordance with the results of previous studies [7]. Specifically, 75% of the HF in which *Legionella* was found in the air by at least one of the culture-based methods reported the highest daily mean values of *Legionella* in water.

Considering only the PG, we found a disagreement between the results obtained by either culture or molecular-based investigations at different sampling hours. This discrepancy has previously been highlighted in water, suggesting that a single sampling would not provide a realistic estimation of *Legionella* risk [34]. Even if culture-based investigation is considered the conventional method for air sampling, the present study demonstrates that it underestimates the actual number of viable microorganisms, such as *Legionella*. In fact, this organism is already difficult to isolate from water systems by standard culture-based techniques when biofilms and/or amoebae are present; therefore, it becomes difficult to detect in the air. Standard cultivation methods for the quantification of *Legionella* are time consuming and labor-intensive, and could be affected by stresses arising during aerosolization of microorganisms leading to a loss of their culturability [35]. Currently, rapid and alternative molecular techniques must be used in combination with culture-based techniques to specify and quantify *Legionella* in the air. Molecular methods, especially those based on PCR, have some important advantages such as the ability to provide results in a few hours and to detect all forms of *Legionella* (alive, viable but not culturable, membrane compromised bacteria, etc.) [21]. 

To the best of our knowledge, this is the first study in which real-time PCR was used to analyze liquid collected by Coriolis^®^μ to detect aerosolized *Legionella*, and showed a larger number of positive results with respect to all culture-based methods (*p* < 0.05). These data are consistent with the results of other investigations [36], and suggest the existence of a fraction of *Legionella* that have enough intact DNA to be detected by molecular methods within three hours, but are not able to replicate on agar medium within 10 days. Therefore, real-time PCR could be considered a relevant tool for auto-controls and rapid monitoring of installations at risk of *Legionella* infection (e.g., cooling towers, hot water supply networks), with quantitative results expressed in numbers of *Legionella* cells (GU). However, replacement of culture-based methods by molecular methods is still ongoing because molecular-based methods do not consider cell viability and can therefore overestimate the risk of *Legionella* infection. To address these concerns, real-time PCR could be combined with measurements of viability such us DNA pre-treatment with ethidium monoazide (EMA) viable dye and propidium monoazide (PMA) [21,35,37]. 

## 5. Conclusions

Our results show that detection of *Legionella* in the air cannot be used to replace water sampling because the absence of the microorganism from the air does not necessarily indicate the absence of water contamination. Nevertheless, air sampling may provide useful information for risk assessment. The liquid impingement technique combined with real-time PCR appears to have the greatest capacity for collecting and detection of airborne *Legionella*. Sampling can be improved by varying details of the culture-based investigation (sampler position, time, culture media, buffers, etc.). Indeed, this is necessary to enable a more accurate assessment of the risk posed by airborne *Legionella*. Overall, the results of this study indicate that molecular investigations could provide useful support to future research in cases of negative culture-based results, and to define a standardized sampling protocol.

## Figures and Tables

**Table 1:** *Legionella* from air and water samples by culturable and molecular investigations.

	Air				Water
	Culturable Investigation			Molecular Investigation	Culturable Investigation
Healthcare Facilities (No.)	Active (cfu/m^3^)		Passive (IMA)	Active (GU/100 mL)	(cfu ^b^/L)
	SAS	Coriolis		Coriolis	
1	*Lpn* sg 10 (1)	0	*Lpn* sg 10 (1)	7947.25 ^a^ (4363.5)	*Lpn* sg 10 (11,333.3)
2	0	0	0	0 ^a^ (0)	*Lpn* sg 1 (5500)
3	0	0	0	0 ^a^ (0)	*Lpn* sg 1 (833.3)
4	0	0	0	415.9 ^a^ (333.95)	*Lpn* sg 3 (3166.7)
5	*Lpn* sg 1 (1)	0	*Lpn* sg 1 + 7 (2.25)	839.5 ^a^ (2818.5)	*Lpn* sg 1 + 3 + 7 (27,333.3)
6	0	0	0	0 ^a^ (95.75)	*Lpn* sg 3 (9133.3)
7	*Lpn* sg 1 (1)	0	0	0 ^a^ (27.75)	*Lpn* sg 1 + 6 (6166.7)
8	0	0	0	3942 ^a^ (3444)	*Lpn* sg 3 (5283.3)
9	0	0	0	656.95 ^a^ (2074.4)	*Lpn* sg 3 (4966.7)
10	0	0	0	0 ^a^ (0)	*Lpn* sg 7 (1033.3)
11	0	0	*Lpn* sg 3 (2)	288.032 ^a^ (978.449)	*Lpn* sg 3 (17,566.7)

^a^ median (interquartile range); ^b^ daily mean values; cfu = colony-forming units; IMA = Index Microbial Air; GU = Genomic Units; SAS = Surface Air System.

**Table 2:** *Legionella* air contamination in PG * by different sampling methods.

Healthcare Facilities (No.)	Methods		Sampling							
			1 h	2 h	3 h	4 h	5 h	6 h	7 h	8 h
1	SAS		+	−	−	−	−	−	+	−
	Settle Plates		+	−	−	−	−	−	+	−
	CORIOLIS	Culture	−	−	−	−	−	−	−	−
		qPCR Lpn	+	+	+	+	+	+	+	+
		qPCR Lspp	+	+	+	+	+	+	+	+
5	SAS		−	+	−	−	−	−	−	−
	Settle Plates		+	+	+	−	+	+	−	−
	CORIOLIS	Culture	−	−	−	−	−	−	−	−
		qPCR Lpn	−	−	+	−	−	−	−	−
		qPCR Lspp	+	+	+	+	+	+	−	+
7	SAS		−	+	−	−	−	−	−	−
	Settle Plates		−	−	−	−	−	−	−	−
	CORIOLIS	Culture	−	−	−	−	−	−	−	−
		qPCR Lpn	+	−	−	−	+	+	+	+
		qPCR Lspp	+	+	−	−	+	+	+	+
11	SAS		−	−	−	−	−	−	−	−
	Settle Plates		+	−	−	−	−	−	−	−
	CORIOLIS	Culture	−	−	−	−	−	−	−	−
		qPCR Lpn	−	−	−	+	−	−	−	−
		qPCR Lspp	+	+	+	+	+	+	−	+

* positive group by at least one culture-based investigation; + = positive results; − = negative results.

## References

[1] World Health Organization (2007). Legionella and the Prevention of Legionellosis.

[2] Linee Guida per la Prevenzione ed il Controllo Della Legionellosi. http://www.salute.gov.it/imgs/C_17_pubblicazioni_2362_allegato.pdf.

[3] European Centre for Disease Prevention and Control (2015). Legionnaires’ Disease in Europe, 2013.

[4] Istituto Superiore di Sanità (2016). Rapporto Annuale sulla legionellosi in Italia nel 2015.

[5] Correia A.M., Ferreira J.S., Borges V., Nunes A., Gomes B., Capucho R., Gonçalves J., Antunes D.M., Almeida S., Mendes A. (2016). Probable person to person transmission of Legionnaires’ disease. N. Engl. J. Med..

[6] Chang C.W., Hung P.Y. (2012). Methods for detection and quantification of airborne *Legionellae* around cooling towers. Aerosol Sci. Technol..

[7] Pasquarella C., Veronesi L., Castiglia P., Liguori G., Montagna M.T., Napoli C., Rizzetto R., Torre I., Masia M.D., Di Onofrio V. (2010). Italian multicentre study on microbial environmental contamination in dental clinics: A pilot study. Sci. Total Environ..

[8] Chang C.W., Chou F.C. (2011). Methodologies for quantifying culturable, viable, and total *Legionella* pneumophila in indoor air. Indoor Air.

[9] Crimi P., Macrina G., Grieco A., Tinteri C., Copello L., Rebora D., Galli A., Rizzelli R. (2006). Correlation between *Legionella* contamination in water and surrounding air. Infect. Control Hosp. Epidemiol..

[10] Blatny J.M., Reif B.A., Skogan G., Andreassen O., Hoiby E.A., Ask E., Waagen V., Aanonsen D., Aaberge I.S., Caugant D.A. (2008). Tracking airborne *Legionella* and *Legionella* pneumophila at a biological treatment plant. Environ. Sci. Technol..

[11] ISO 14698-1:2003 Cleanrooms and Associated Controlled Environments: Biocontamination Control; Part 1: General Principles and Methods. https://www.iso.org/standard/25015.html.

[12] Pasquarella C., Pitzurra O., Savino A. (2000). The index of microbial air contamination. J. Hosp. Infect..

[13] Pasquarella C., Albertini R., Dall’Aglio P., Saccani E., Sansebastiano G.E., Signorelli C. (2008). Air microbial sampling: The state of the art. Ig Sanità Pubblica.

[14] Pasquarella C., Veronesi L., Napoli C., Castiglia P., Liguori G., Rizzetto R., Torre I., Righi E., Farruggia F., Tesauro M. (2012). Microbial environmental contamination in Italian dental clinics: A multicenter study yielding recommendations for standardized sampling methods and threshold values. Sci. Total Environ..

[15] Pasquarella C., Vitali P., Saccani E., Manotti P., Boccuni C., Ugolotti M., Signorelli C., Mariotti F., Sansebastiano G.E., Albertini R. (2012). Microbial air monitoring in operating theatres: Experience at the University Hospital of Parma. J. Hosp. Infect..

[16] Montagna M.T., De Giglio O., Napoli C., Cannova L., Cristina M.L., Deriu M.G., Delia S.A., Giuliano A., Guida M., Lagana P. (2014). *Legionella* spp. contamination in indoor air: Preliminary results of an Italian multicenter study. Epidemiol. Prev..

[17] Montagna M.T., Cristina M.L., De Giglio O., Spagnolo A.M., Napoli C., Cannova L., Deriu M.G., Delia S.A., Giuliano A., Guida M. (2016). Serological and molecular identification of *Legionella* spp. isolated from water and surrounding air samples in Italian healthcare facilities. Environ. Res..

[18] Montagna M.T., De Giglio O., Cristina M.L., Albertini R., Pasquarella C., GISIO, AIA and SIMPIOS Working Groups (2017). Legionella Indoor Air Contamination in Health Care Environments. SpringerBriefs Public Health.

[19] An H.R., Mainelis G., White L. (2006). Development and calibration of real-time PCR for quantification of airborne microorganisms in air samples. Atmos. Environ..

[20] Deloge-Abarkan M., Ha T.L., Robine E., Zmirou-Naviera D., Mathieu L. (2007). Detection of airborne *Legionella* while showering using liquid impingement and fluorescent in situ hybridization (FISH). J. Environ. Monit..

[21] Lee J.V., Lai S., Exner M., Lenz J., Gaia V., Casati S., Hartemann P., Lück C., Pangon B., Ricci M.L. (2011). An international trial of quantitative PCR for monitoring *Legionella* in artificial water systems. J. Appl. Microbiol..

[22] Langer V., Hartmann G., Niessner R., Seidel M. (2012). Rapid quantification of bioaerosols containing L. pneumophila by Coriolis^®^ µ air sampler and chemiluminescence antibody microarrays. J. Aerosol. Sci..

[23] Montagna M.T., Napoli C., Tatò D., Spilotros G., Barbuti G., Barbuti S. (2006). Clinical-environmental surveillance of legionellosis: An experience in Southern Italy. Eur. J. Epidemiol..

[24] Napoli C., Fasano F., Iatta R., Barbuti G., Cuna T., Montagna M.T. (2010). *Legionella* spp. and legionellosis in south eastern Italy: Disease epidemiology and environmental surveillance in community and healthcare facilities. BMC Public Health.

[25] Napoli C., Marcotrigiano V., Montagna M.T. (2012). Air sampling procedures to evaluate microbial contamination: A comparison between active and passive methods in operating theatres. BMC Public Health.

[26] Napoli C., Tafuri S., Montenegro L., Cassano M., Notarnicola A., Lattarulo S., Montagna M.T., Moretti B. (2012). Air sampling methods to evaluate microbial contamination in operating theatres: Results of a comparative study in an orthopaedics department. J. Hosp. Infect..

[27] Carvalho E., Sindt C., Verdier A., Galan C., O’Donoghue L., Parks S., Thibaudon M. (2008). Performance of the Coriolis air sampler, a high-volume aerosol-collection system for quantification of airborne spores and pollen grains. Aerobiologia.

[28] Ahmed El Metwaly M.F., Schulz J., Hartung J. (2013). Air samplings in a Campyobacter jejuni positive laying hen flock. Ann. Agric. Environ. Med..

[29] Gaia V., Fry N.K., Afshar B., Lück P.C., Meugnier H., Etienne J., Peduzzi R., Harrison T.G. (2005). Consensus sequence-based scheme for epidemiological typing of clinical and environmental isolates of *Legionella* pneumophila. J. Clin. Microbiol..

[30] Ratzow S., Gaia V., Helbig J.H., Fry N.K., Lück P.C. (2007). Addition of neuA, the gene encoding N-acyl neuraminate cytidylyl transferase, increases the discriminatory ability of the consensus sequence-based scheme for typing *Legionella* pneumophila serogroup1 strains. J. Clin. Microbiol..

[31] Farhat C., Mentasti M., Jacobs E., Fry N.K., Lück C. (2011). The N-acyl neuraminate cytidyl transferase gene, neuA, isheterogenous in *Legionella* pneumophila strains but can be used as a marker for epidemiological typing in the consensus sequence-based typing scheme. J. Clin. Microbiol..

[32] Conza L., Casati S., Gaia V. (2013). Detection limits of *Legionella* pneumophila in environmental samples after co-culture with Acanthamoeba polyphaga. BMC Microbiol..

[33] Ishimatsu S., Miyamoto H., Hori H., Tanaka I., Yoshida S.I. (2001). Sampling and Detection of *Legionella* pneumophila Aerosols Generated from an Industrial Cooling Tower. Ann. Occup. Hyg..

[34] Napoli C., Iatta R., Fasano F., Marsico T., Montagna M.T. (2009). Variable bacterial load of *Legionella* spp. in a hospital water system. Sci. Total Environ..

[35] Scaturro M., Fontana S., Dell’eva I., Helfer F., Marchio M., Stefanetti M.V., Cavallaro M., Miglietta M., Montagna M.T., De Giglio O. (2016). A multicenter study of viable PCR using propidium monoazide to detect *Legionella* in water samples. Diagn. Microbiol. Infect. Dis..

[36] Mathieu L., Robine E., Deloge-Abarkan M., Ritoux S., Pauly D., Hartemann P., Zmirou-Navier D.J. (2006). *Legionella* bacteria in aerosols: Sampling and analytical approaches used during the legionnaires disease outbreak in Pas-de-Calais. Infect. Dis..

[37] Mansi A., Amori I., Marchesi I., Marcelloni A.M., Proietto A.R., Ferranti G., Magini V., Valeriani F., Borella P. (2014). *Legionella* spp. survival after different disinfection procedures: Comparison between conventional culture, qPCR and EMA-qPCR. Microchem. J..

